# Successful treatment of primary delusional parasitosis with paroxetine: A case report and narrative review

**DOI:** 10.1192/j.eurpsy.2021.851

**Published:** 2021-08-13

**Authors:** L. Delgado, A. González-Rodríguez, A. Alvarez Pedrero, A. Guàrdia, G.F. Fucho, M.V. Seeman, S. Acebillo, J.A. Monreal, J. Labad, D. Palao Vidal

**Affiliations:** 1 Mental Health, Parc Tauli University Hospital, Sabadell, Spain; 2 Mental Health, Parc Taulí University Hospital. Autonomous University of Barcelona (UAB). I3PT, Sabadell, Spain; 3 Psychiatry, University of Toronto, Toronto, Canada; 4 Mental Health, Hospital of Mataró. Consorci Sanitari del Maresme. CIBERSAM., Mataró, Spain; 5 Department Of Mental Health, Parc Taulí University Hospital. Autonomous University of Barcelona (UAB). I3PT. CIBERSAM, Sabadell, Spain

**Keywords:** Delusional parasitosis, Antidepressants, psychosis, Antipsychotics

## Abstract

**Introduction:**

Antipsychotics have been classically considered the treatment of choice for delusional disorder (DD) and antidepressant medications have been restricted to patients with comorbid depression.

**Objectives:**

Our aim is to describe the case of a patient with DD with delusions of parasitosis, who responded to paroxetine as monotherapy. We also aimed to review the recent literature on the potential use of antidepressants as the main treatment for somatic type DD.

**Methods:**

After the case report, we present a narrative review on the use of antidepressants in DD, somatic type (DSM-criteria) by using PubMed database from inception until 2020.

**Results:**

Case: 74 year-old woman without previous psychiatric diagnosis who suffered from long-term cutaneous and vulvar pruritus. She was referred to psychiatry from dermatology to assess thought content and sensoperceptive disturbances. In the past, she had received unsuccessful treatment with antihistamines. The patient brought a collection of “the identified parasite” (matchbox sign) to our first appointment. On assessment, she was diagnosed with DD with delusions of parasitosis. Risperidone 1mg/day was poorly tolerated (excessive sedation). She refused further antipsychotic treatment, so we started paroxetine up to 20mg/day. The patient went into total remission of her pruritus and delusions of parasitosis. Review. In line with our case, 6 studies reported on the successful use of antidepressants as monotherapy for DD, somatic type. Most of studies report the successful use of an antipsychotic/antidepressant combination (case-series, case reports).

**Conclusions:**

Although antipsychotics are the treatment of choice, antidepressant medications may be an effective alternative in somatic type DD when patients refuse antipsychotics.

